# Health and the Myrmidons

**DOI:** 10.3201/eid1805.AC1805

**Published:** 2012-05

**Authors:** Polyxeni Potter

**Affiliations:** Centers for Disease Control and Prevention, Atlanta, Georgia, USA

**Keywords:** art science connection, emerging infectious diseases, art and medicine, Salvador Dalí, Health and the myrmidons, Daddy Longlegs of the Evening―Hope!, Spanish art, viruses, about the cover

**Figure:**
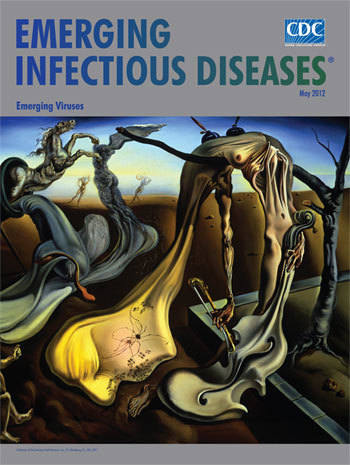
**Salvador Dalí (1904–1989) *Daddy Longlegs of the Evening―Hope!* (1940) Oil on canvas (25.4 cm × 50.8 cm)** Fundación Gala-Salvador Dalí, (Artists Rights Society), 2011. Collection of the Salvador Dalí Museum, Inc., St. Petersburg, FL, USA, 2011

“I’ll be a genius, and the world will admire me,” Salvador Dalí wrote in his diary at age 15. These confident words marked a journey of greatness started during childhood, in Figueres, Spain. In this rural town in Catalonia, steeped in artistic heritage, he started to read Voltaire, Nietzsche, Kant, Spinoza, and Descartes, and these philosophers’ notions on the nature of reality created in his mind an abiding sense of purpose.

Much of Dalí’s boyhood was spent in his parents’ home at the coastal village of Cadaqués near Port Lligat, where he later made his own home. Rocks from the local beach found their way into many of his works. His early art education benefited from frequent visits with the family of artist Ramón Pichot and from studies at the Municipal School of Drawing in Figueres under engraver Juan Nuñez. Dalí’s earliest surviving works date from this period, during which he also wrote copiously and showed interest in cubism. Soon he entered the famed Academy of San Fernando in Madrid, where he met poet Federico García Lorca, who later published an ode in his honor, “O Salvador Dalí of the olive-colored voice / I do not praise your halting adolescent brush / or your pigments that flirt with the pigments of your times / but I laud your longing for eternity with limits.”

As a student in Madrid, Dalí got to know the Prado Museum and the cubist works of Picasso, Georges Braque, Juan Gris, and the metaphysical paintings of Giorgio de Chirico. Encouraged by the attention of his peers and association with Lorca, he ventured outside the restrictions and requirements of the school. Increasingly disenchanted, he was suspended and later expelled by the academy, as he was at some point to be expelled from the surrealist movement, again for unwillingness to color inside the lines.

In a long period of experimentation also marked by personal notoriety, Dalí took his work in many directions, from a strictly academic style to cubism. An astonishing draftsman, he drew from nature, the imagination, or classical tradition as he searched for a distinctive style. He traveled to Paris where he eagerly embraced surrealist concepts. A solo exhibition there in 1929 brought a glowing review from poet André Breton, psychiatrist and head of the surrealist group.

Surrealists, Dalí among them, wanted to shake up comfortable middle class values, preferring to depict life as lived by the mind. This approach, with its probing of the unconscious, dreams, memories, and psychological associations, formed the foundation of new art rooted in paradox and contradictions rather than mimesis.

*Daddy Longlegs of the Evening―Hope!* on this month’s cover, was inspired by war, the carnage of which Dalí experienced from close up. The Spanish Civil War took him to Paris. Shortly after his departure, the family home in Cadaqués was bombed, his own place at Port Lligat ransacked and destroyed. His friend Lorca was executed; his sister, imprisoned and tortured. Chased to Italy and then again to Paris, he lived there until the outbreak of World War II, when once more he had to flee. His work of this time grew dark and intense, evoking tragedy, melancholy, and confusion. In 1940, he emigrated to the United States, where he was to live for 8 years.

In *Daddy Longlegs of the Evening―Hope!* the landscape is denuded. On the right side, an olive tree, the perennial emblem of peace, is bare of leaves. On the horizon, two human figures are engaged in some macabre dance, their elongated shapes and ballooned sleeves mocking similar forms in the foreground. Evening shadows cast a mournful look over the scene. In the center, Dalí places his own image, a melting face drawn in the outline of his familiar beach rocks and joined to Creativity, a hollow figure draped over the tree that once nourished it.

Literary and music references, two inkwells and a prominent cello, complete the hollow figure, offering clues about Dali’s state of mind. On the upper left corner, the cannon from Giorgio de Chirico’s painting *The Philosopher’s Conquest* (1914) is held by a crutch, the cannonball melting into distorted body parts, lifeless fluids, a spent sperm-like form. Out of this unlikely background emerges a bandaged *Nike of Samothrace*, ill-defined and threatened by a decayed horse of the apocalypse leaping from the cannon. In the lower left corner, a winged putto, messenger of love and artistic pursuits, weeps at the spectacle. But despite the devastation, a symbol of hope appears on the artist’s head: daddy longlegs, a token of good luck, amidst swarming ants.

The Myrmidons, ant-men (from *μύρμηξ* [*murmex*] “ant”) mentioned in The Iliad and described in some detail by Ovid in the Metamorphoses, were a prolific warrior race patterned after ants from whom they allegedly descended by divine transformation. The term, which has survived with various connotations, still at times denotes mindless masses or “hired ruffians,” who make up for unquestioning loyalty with sheer numbers. The term also still evokes somehow the ants of origin―the same plentiful arthropods Dalí sprinkled liberally in his paintings, along with flies and other insects―their glistening bodies clustered around the edges, foreshadowing decomposition.

“Beautiful as the chance meeting on a dissecting table of a sewing machine and an umbrella,” is how revelatory juxtapositions were viewed by the surrealists. And the marvelous was to be detected in the everyday, the discarded, the coincidental, and the unnoticed. These ideas drove surrealism and, no longer incongruous or farfetched, now find their way in all aspects of life and no less in science.

Among health threats, viruses are like the Myrmidons in their sheer numbers. Even though several thousand have been identified, the large masses remain at large. With each new identification, the public health burden increases. And this is where Dalí would insert daddy longlegs. For with each identification, the opportunity also arises for new vaccines or other prevention strategies and effective treatments, as in the case of many known viruses, including the formidable HIV.

Like a surrealist painting, emergence of viruses around the globe features realities that by all appearances have nothing to link them, often in settings that by all appearances are not linked. In this issue alone, a new strain of Andes virus associated with fatal human infection was found in central Bolivia; and a new human adenovirus, in Bangladesh. Adenovirus type 7 is emerging in Malaysia. A variant West Nile virus strain, most related to the indigenous Kunjin, was characterized in Australia. Lymphocytic choriomeningitis virus–associated meningitis is reported in southern Spain; and hepatitis E virus infection, in solid organ transplant recipients in the Netherlands. The chance meeting on a “dissecting table” still applies, and the marvelous resides in identifying and controlling the Myrmidons, one by one.
